# Urinary tobacco-specific nitrosamine 4-(methylnitrosamino)-1-(3-pyridyl)-1-butanol (NNAL) and cognitive functioning in older adults: The National Health and Nutrition Examination Survey 2013–2014

**DOI:** 10.18332/tid/162368

**Published:** 2023-05-25

**Authors:** Song Ge, Weixia Ma, Zhe Qu, Xingmei Zhu, Zijing Chen, Xuechun Lin, Zhenmei Fu

**Affiliations:** 1 Department of Natural Sciences, College of Sciences and Technology, University of Houston-Downtown, Houston, United States; 2 Department of Pulmonary and Critical Care Medicine, Shandong Provincial Hospital Affiliated to Shandong First Medical University, Jinan, China; 3 School of Nursing, Xuzhou Medical University, Xuzhou, China; 4 Yaxin School of Nursing, Wuhan Institute of Design and Science, Wuhan, China; 5 Chinese Nursing Association, Beijing, China; 6 Department of Nutrition and Food Hygiene, Hubei Key Laboratory of Food Nutrition and Safety, Ministry of Education Key Laboratory of Environment and Health, School of Public Health, Tongji Medical College, Huazhong University of Science and Technology, Wuhan, China; 7 Department of Radiology, Shandong Provincial Hospital Affiliated to Shandong First Medical University, Jinan, China

**Keywords:** tobacco-specific nitrosamines, cognitive function, older adults, NHANES, smoking

## Abstract

**INTRODUCTION:**

Tobacco contains carcinogens called tobacco-specific nitrosamines. Among the tobacco-specific nitrosamines, is nicotine-derived nitrosamine ketone (NNK) which produces the metabolite 4-(methylnitrosamino)-1-(3-pyridyl)-1-butanol (NNAL). We aimed to examine the association between urinary tobacco-specific NNAL and cognitive functioning among older adults.

**METHODS:**

A total of 1673 older adults aged ≥60 years from the National Health and Nutrition Examination Survey 2013–2014 were included. Urinary tobacco-specific NNAL was analyzed in the laboratory. Cognitive functioning was measured using the Consortium to Establish a Registry for Alzheimer’s Disease Word Learning subtest (CERAD-WL) immediate and delayed memory tests, the Animal Fluency test (AFT), and the Digit Symbol Substitution Test (DSST). Test-specific and global cognition z-scores were calculated based on means and standard deviations of the cognitive test scores. Multivariable linear regression models were constructed to examine the independent association between quartiles of urinary tobacco-specific NNAL and cognitive test-specific and global cognition z-scores controlling for age, sex, race/ethnicity, education level, depressive symptoms, body mass index, systolic blood pressure, urinary creatinine, hypertension, diabetes, alcohol use, and smoking status.

**RESULTS:**

About half of the participants (mean age 69.8 years) were female (52.1%), non-Hispanic White (48.3%), and completed some college and above (49.7%). Multivariable linear regression results showed that participants in the 4th quartile (highest quartile) of urinary NNAL, compared with those in the 1st quartile (lowest quartile), had lower DSST z-scores (β= -0.19; 95% CI: -0.34 – -0.04).

**CONCLUSIONS:**

Tobacco-specific NNAL was negatively associated with processing speed, sustained attention, and working memory in older adults.

## INTRODUCTION

The prevalent Alzheimer’s disease and related dementia (ADRD) worldwide poses a severe threat to families, communities, and healthcare systems. The risk of ADRD increases with age^1^. Thus, with population aging and prolonged life expectancy, the number of people living with dementia has more than doubled between 1990 and 2016^2^. ADRD has become a leading cause of mortality and morbidity among older adults worldwide, accounting for 33.1 million disability-adjusted life years lost in 2019^3^. One of the main approaches to reducing the burden of ADRD is to identify and intervene in modifiable risk factors of ADRD before it occurs, since ADRD is currently incurable. According to a recent study, addressing modifiable risk factors has led to a decline in age-specific dementia incidences in the US^4^.

A recent study showed that although the prevalence of cigarette smoking in the US has declined over the past decades, it is still common in older adults, with 9% of older adults aged ≥65 years being current smokers^5^. In addition, many older adults are exposed to tobacco through secondhand tobacco smoke^6^. Many studies have examined the relationship between tobacco smoking and cognitive functioning in older adults. While most studies have found harmful cognitive effects of tobacco smoking^7,8^, some studies had an opposite finding^9^. Thus, this question needs to be further elucidated. In addition, most existing studies used participants’ self-report pack-years^9^ or serum/urinary cotinine level as a biomarker for tobacco exposure^10^. However, cotinine, a major proximate metabolite of nicotine, has a short half-life (16 h) and can only indicate very recent exposure to tobacco. Tobacco and tobacco smoke contain a class of carcinogens called tobacco-specific nitrosamines formed during tobacco curing and burning^10^. A metabolite of nicotine-derived nitrosamine ketone (NNK) is 4-(methylnitrosamino)-1-(3-pyridyl)-1-Butanol (NNAL)^11^. To our knowledge, no study has examined the relationship between cigarette exposure using urinary tobacco-specific NNAL and cognitive functioning. A previous study has found that with a longer half-life (10–16 days), tobacco-specific NNAL is probably a better biomarker of cumulative cigarette exposure over time compared with cotinine and can differentiate active smoking and passive smoking well^10^.

In this study, we utilized data from the National Health and Nutrition Examination Study^12^ to examine the association between urinary tobacco-specific NNAL and cognitive functioning in a nationally representative sample of US older adults. Therefore, this study has good generalizability. The findings of this study will help us elucidate the relationship between tobacco exposure and cognitive functioning in the growing proportion of older adults worldwide.

## METHODS

### The parent study design and recruitment

The National Center for Health Statistics of the Centers for Disease Control and Prevention (CDC) conducts the NHANES, a continuous cross-sectional survey of civilian, non-institutionalized adults and children in the US every two years^13^. The NHANES is a nationally representative sample of the United States. Participants are recruited nationwide for each two-year cycle using a complex, multistage probability strategy involving a group of census blocks or area segments within clusters of census blocks^14^. Face-to-face interviews at participants’ homes and health examinations at mobile centers with specialized equipment are used to assess participants’ sociodemographic, health, and nutritional status. Each participant aged ≥6 years provides urine samples for analysis of urinary NNAL. For this analysis, we included participants in NHANES 2013–2014 aged ≥60 years with available information on urinary tobacco-specific NNAL and cognitive test performance. A total of 9813 individuals took part in the NHANES 2013–2014 survey. We excluded those who were aged <60 years (n=8028) or had missing data on urinary NNAL (n=112). Finally, a total of 1673 participants aged ≥60 years were included in the analysis.

### Measures

*Independent variable: Quartile of urinary NNAL (free NNAL plus NNAL-glucuronide, ng/mL)*

NNK [4-(methylnitrosamino)-1-(3-pyridyl)-1-butanone] is found extensively in tobacco and tobacco smoke. NNK is quickly transformed into its metabolite, NNAL [4-(methylnitrosamino)-1-(3-pyridyl)-1-butanol], in the body of a smoker. NNAL may also be present in the urine as NNAL-Glucuronide (NNAL-N-Glucuronice and NNAL-O-Glucuronide) as it is further conjugated with glucuronic acid^10^. The sum of free NNAL and NNAL-glucuronide in the urine (ng/mL) was categorized into four groups based on its quartile. The quartile variable on urinary NNAL is used as the independent variable of this study.

Participants’ urine samples were collected during physical examinations, aliquoted, and kept frozen at -20°C until they arrived at the Division of Laboratory Sciences, National Center for Environmental Health, and CDC for analysis. An isotope-dilution high-performance liquid chromatography/electrospray ionization tandem mass spectrometry (ID HPLC-ESI MS/MS) was used to measure tobacco-specific NNAL in urine. A ^13^C_6_-labeled NNAL internal standard was used to spike 5 mL urine samples, which were then hydrolyzed with glucuronidase for at least 24 h. After that, high-performance liquid chromatography atmospheric-pressure ionization tandem mass spectrometry (HPLC-API MS/MS) was used to process and analyze the hydrolysate. The peak area ratio of the internal standard to the analyte was used to determine the amount of NNAL. The detailed method has been published on the NHANES website^12^.

In each analytical run, a blank and two quality-control pools were examined. The Division of Laboratory Sciences, National Center for Environmental Health, CDC reported accurate and precise results according to its quality control/quality assurance program^15^. Relying on the variance from the repeated analysis of a small, spiked urine sample (2 pg/mL), this method for measuring NNAL has a lower limit of detection (LLOD) at 0.6 pg/mL. For results below LLOD, an imputed fill value was used. This value was computed as LLOD/√2. Researchers have demonstrated that NNAL is stable in urine during long-term storage at -70°C for no less than several years^16^.

*Dependent variable: Cognitive functioning*

The Consortium to Establish a Registry for Alzheimer’s Disease Word Learning subtest (CERAD-WL), the Animal Fluency test (AFT), and the Digit Symbol Substitution Test (DSST) were used to assess participants’ cognitive functioning.

Using an immediate memory test and a delayed memory test after three consecutive immediate learning trials, the CERAD-WL evaluated participants’ ability to acquire new verbal knowledge^17^. In the immediate memory test, participants were asked to read ten random words shown as large, bolded letters on a computer monitor aloud in each of three learning trials, one at a time. After the ten words were presented, participants needed to remember and recall as many words as they could immediately. The sequence of these ten words was different in each trial. For each trial, the maximum score is ten. Therefore, the total score range of three trials was 0 to 30, representing the participant’s immediate memory score.

After finishing the other two cognitive tests (the DSST and the AFT), participants were instructed to recall as many words from the same ten-word list as they could. This formed their delayed memory test. The participant’s delayed memory test score consisted of the number of correct words they could recall and ranged from 0 to 10.

The AFT evaluated participants’ language fluency and executive function^18^. Participants were informed to name as many animals as they could in 60 seconds. Each animal called counted one score.

Participants’ processing speed, sustained attention, and working memory was measured by the DSST^19^. A paper form with a top-mounted key that had nine numbers and paired symbols was used to administer this test. Participants had two minutes to copy the symptoms to the 133 boxes next to the numbers containing the corresponding symbols. The total number of right matches determined the score with the possible score range between 0 and 133.

### Covariates

To account for potential confounding factors between urinary tobacco-specific NNAL and cognitive functioning, we controlled the following covariates in the analysis, including age (years), sex (male or female), race/ethnicity (Mexican American, other Hispanic, non-Hispanic White, or non-Hispanic Black), education level (below high school, high school graduate, or some college or higher), depressive symptoms, body mass index (<18.5, 18.5–24.9, 25–29.9, or ≥30 kg/m^2^), systolic blood pressure (mmHg), urinary creatinine (mg/dL), hypertension (yes or no), diabetes (yes or no), alcohol use (current drinker or not) and smoking status (current smoker or not). All the information was obtained from face-to-face interviews or health examinations. The Patient Health Questionnaire (PHQ-9) score was used to indicate depressive symptoms^20^.

### Statistical analysis

We summarized the characteristics of participants using mean and standard deviation (SD) for continuous variables and number and percentage for categorical variables.

The CERAD-WL immediate memory, the CERAD-WL delayed memory, the AFT, and the DSST z-scores were calculated using sample means and SDs of the cognitive test scores. We determined cognitive test-specific z-scores to have a mean of 0 and a variation of 1 because the cognitive tests employed in this study were based on several scales. To generate a global cognitive z-score, we computed an arithmetic mean of the four z-scores from various cognitive tests. Multivariable regression models were constructed to examine the relationship between quartiles of urinary tobacco-specific NNAL (Ref. 1st quartile, the lowest quartile) and test-specific and global cognition z-scores, controlling for age, sex, race/ethnicity, education level, depressive symptoms, body mass index, systolic blood pressure, urinary creatinine, hypertension, diabetes, alcohol use, and smoking status. We considered a 95% confidence interval (CI) excluding zero as statistically significant. All analyses were performed using SPSS 25.0.

## RESULTS

The characteristics of the excluded participants due to missing data (n=112) are summarized in Supplementary file Table 1. Compared with the included participants, the excluded participants were more likely to be older, other Hispanic and other race/ethnicity, and had more depressive symptoms, lower CERAD W-L immediate recall, lower CERAD W-L immediate recall, lower AFT, and lower DSST scores.

The characteristics of the study population are presented in Table 1. The 1673 participants had a mean age of 69.8 years (SD=6.8). About half of the participants were female (52.1%), non-Hispanic White (48.3%), completed some college or higher (49.7%), had a BMI ≥30 kg/m^2^ (36.1%), had a mean systolic blood pressure of 116.5 mmHg, and had hypertension (63.2%). Their mean urinary creatinine was 106.5 mg/dL (SD=68.2). Twenty-three percent of the participants had diabetes. The participants’ mean urinary NNAL (ng/mL) was 0.8749 (SD=0.5261), ranging from 0.0004 to 13.2000. Their mean CERAD-WL immediate memory score was 19.4 (SD=4.9), mean CERAD-WL delayed memory score 6.1 (SD=2.4), mean AFT score 16.6 (SD=5.5), and mean DSST score 46.4 (SD=17.2).

**Table 1:** Characteristics of the participants by tobacco-specific nitrosamine quartile: the National Health and Nutrition Examination Survey 2013–2014

*Variables*	*Quartile 1 ≤0.0004 ng/mL (N=736) n (%)*	*Quartile 2 0.0004–0.0007 ng/mL (N=120) n (%)*	*Quartile 3 0.0007–0.0048 ng/mL (N=401) n (%)*	*Quartile 4 >0.0048 ng/mL (N=416) n (%)*	*Total (N=1673) n (%)*
**Age** (years), mean ± SD	70.8 ± 6.9	69.5 ± 6.5	69.5 ± 6.8	68.1 ± 6.2	69.8 ± 6.8
**Sex**					
Male	322 (43.8)	59 (49.2)	190 (47.4)	231 (55.5)	802 (47.9)
Female	414 (56.3)	61 (50.8)	211 (52.6)	185 (44.5)	871 (52.1)
**Race/ethnicity**					
Mexican American	77 (10.5)	13 (10.8)	61 (15.2)	47 (11.3)	198 (11.8)
Other Hispanic	60 (8.2)	11 (9.2)	36 (9.0)	38 (9.1)	145 (8.7)
Non-Hispanic White	418 (56.8)	56 (46.7)	171 (42.6)	163 (39.2)	808 (48.3)
Non-Hispanic Black	94 (12.8)	22 (18.3)	91 (22.7)	139 (33.4)	346 (20.7)
Other	87 (11.8)	18 (15.0)	42 (10.5)	29 (7.0)	176 (10.5)
**Education level**					
Below high school	139 (18.9)	24 (20.0)	123 (30.7)	155 (37.3)	441 (26.3)
High school graduate	161 (21.9)	30 (25.0)	88 (21.9)	120 (28.8)	399 (23.8)
Some college or higher	435 (59.1)	66 (55.0)	190 (47.4)	140 (33.7)	831 (49.7)
**Health status**					
Depressive symptoms, mean ± SD	3.3 ± 4.5	3.7 ± 5.1	3.9 ± 5.1	4.6 ± 5.4	3.8 ± 4.9
**BMI** (kg/m^2^)					
<18.5	9 (1.2)	1 (0.8)	3 (0.7)	16 (3.8)	29 (1.7)
18.5–24.9	191 (26.0)	27 (22.5)	81 (20.2)	116 (27.9)	415 (24.8)
25.0–29.9	267 (36.3)	42 (35.0)	146 (36.4)	145 (34.9)	600 (35.9)
≥30	255 (34.5)	47 (39.2)	168 (41.9)	134 (32.2)	604 (36.1)
Systolic blood pressure (mmHg), mean ± SD	116.8 ± 16.0	115.2 ± 16.9	115.2 ± 15.1	117.7 ± 17.1	116.5 ± 16.2
Urinary creatinine (mg/dL), mean ± SD	87.3 ± 55.6	118.1 ± 65.6	116.4 ± 65.9	127.4 ± 81.7	106.5 ± 68.3
Hypertension	472 (64.1)	74 (61.7)	265 (66.1)	246 (59.1)	1057 (63.2)
Diabetes	155 (21.1)	30 (25.0)	106 (26.4)	94 (22.6)	385 (23.0)
Current drinker	77 (10.5)	9 (7.5)	68 (17.0)	100 (24.0)	254 (15.2)
Current smoker	2 (0.3)	0 (0)	1 (0.2)	196 (47.1)	199 (11.9)
CERAD W-L immediate recall, mean ± SD	19.8 ± 4.8	19.4 ± 5.1	19.1 ± 5.2	19.0 ± 4.5	19.4 ± 4.9
CERAD W-L delayed recall, mean ± SD	6.3 ± 2.4	6.1 ± 2.5	6.0 ± 2.3	5.9 ± 2.3	6.1 ± 2.4
Animal Fluency Test, mean ± SD	17.1 ± 5.3	17.0 ± 5.9	16.0 ± 5.5	16.0 ± 5.8	16.6 ± 5.5
Digit Symbol Substitution Test, mean ± SD	49.4 ± 17.2	49.4 ± 16.7	44.5 ± 17.0	41.8 ± 16.3	46.4 ± 17.2

The means and 95% CIs of the cognitive test-specific z-scores by tobacco-specific nitrosamine quartiles are shown in Table 2. For participants in 1st quartile (the lowest quartile) of tobacco-specific nitrosamine, their mean z-score of CERAD W-L immediate recall, CERAD W-L delayed recall, AFT and DSST was 0.08 (95% CI: -1.87–2.03), 0.08 (95% CI: -1.91–2.06), 0.09 (95% CI: -1.80–1.98), and 0.18 (95% CI: -1.79–2.14), respectively. For participants in the 2nd quartile of tobacco-specific nitrosamine, their mean z-score of CERAD W-L immediate recall, CERAD W-L delayed recall, AFT and DSST was -0.001 (95% CI: -2.04–2.04), 0.002 (95% CI: -2.06–2.07), 0.008 (95% CI: -2.00–2.17), and 0.17 (95% CI: -1.73–2.08), respectively. Among participants in the 3rd quartile, their mean z-score of CERAD W-L immediate recall, CERAD W-L delayed recall, AFT and DSST was -0.05 (95% CI: -2.12–2.02), -0.06 (95% CI: -1.98–1.87), -0.10 (95% CI: -2.03–1.84), and -0.11 (95% CI: -2.05–1.83), respectively. The mean z-score of CERAD W-L immediate recall, CERAD W-L delayed recall, AFT and DSST was -0.09 (95% CI: -1.92–1.74), -0.08 (95% CI: -1.98–1.82), -0.10 (95% CI: -2.13–1.94), -0.27 (95% CI: -2.12–1.59), respectively, among participants in the 4th quartile (the highest quartile). The mean global cognition z-score of quartiles 1 to 4 was 0.12 (95% CI: -1.84–2.08), 0.09 (95% CI: -1.92–2.09), -0.08 (95% CI: -2.04–1.87), and -0.17 (95% CI: -2.06–1.72), respectively.

**Table 2:** Participants’ cognitive specific test and global cognition z-scores and 95% confidence intervals by tobacco-specific nitrosamine quartile

	*Quartile 1 ≤0.0004 ng/mL (N=736)*	*Quartile 2 0.0004–0.0007 ng/mL (N=120)*	*Quartile 3 0.0007–0.0048 ng/mL (N=401)*	*Quartile 4 >0.0048 ng/mL (N=416)*
CERAD W-L immediate recall	0.08 (-1.87–2.03)	-0.001 (-2.04–2.04)	-0.05 (-2.12–2.02)	-0.09 (-1.92–1.74)
CERAD W-L delayed recall	0.08 (-1.91–2.06)	0.002 (-2.06–2.07)	-0.06 (-1.98–1.87)	-0.08 (-1.98–1.82)
Animal Fluency Test	0.09 (-1.80–1.98)	0.08 (-2.00–2.17)	-0.10 (-2.03–1.84)	-0.10 (-2.13–1.94)
Digit Symbol Substitution Test	0.18 (-1.79–2.14)	0.17 (-1.73–2.08)	-0.11 (-2.05–1.83)	-0.27 (-2.12–1.59)
Global cognition	0.12 (-1.84–2.08)	0.09 (-1.92–2.09)	-0.08 (-2.04–1.87)	-0.17 (-2.06–1.72)

Multivariable linear regression results (Table 3) showed that participants in the 4th quartile (highest quartile) of urinary NNAL, compared with those in the 1st quartile (lowest quartile), had lower DSST z-scores (β= -0.19; 95% CI: -0.34 – -0.04).

**Table 3:** Linear regression results on the independent associations of tobacco-specific nitrosamine quartile with cognitive specific test and global cognition z-scores among older adults^a^ [Ref. Quartile 1 (≤0.0004 ng/mL)]

	*Quartile 2 0.0004–0.0007 ng/mL*	*Quartile 3 0.0007–0.0048 ng/mL*	*Quartile 4 >0.0048 ng/mL*
CERAD W-L immediate recall	0.02 (-0.12–0.16)	-0.08 (-0.22–0.06)	-0.08 (-0.25–0.09)
CERAD W-L delayed recall	0.04 (-0.10–0.19)	-0.05 (-0.19–0.10)	-0.02 (-0.19–0.16)
Animal Fluency Test	-0.07 (-0.22–0.07)	-0.14 (-0.29–0.01)	-0.07 (-0.25–0.11)
Digit Symbol Substitution Test	0.10 (-0.02–0.22)	-0.10 (-0.22–0.02)	**-0.19 (-0.34 – -0.04)^b^**
Global cognition	0.05 (-0.08–0.18)	-0.11 (-0.23–0.02)	-0.10 (-0.26–0.06)

a Models were adjusted for age, sex, race/ethnicity, education level, depressive symptoms, body mass index, systolic blood pressure, urinary creatinine, hypertension, diabetes, alcohol use and smoking status.

b Values in bold denote statistical significance (95% confidence interval excluding zero).

## DISCUSSION

In this group of 1673 nationally representative sample of US older adults, higher urinary tobacco-specific NNAL (free NNAL plus NNAL-glucuronide) was independently associated with worse processing speed, sustained attention, and working memory. Although our results still need to be validated by longitudinal studies, they indicate that exposure to tobacco may be associated with worse cognitive functioning in older adults.

A number of published studies have examined the relationship between smoking and cognitive functioning. In the Whitehall II Cohort Study, middle-aged male smokers showed a faster decline in global cognition and executive function than non-smokers. However, there were no negative effects on cognitive decline in ex-smokers who had quit smoking for at least 10 years^21^. In another observational web-based cohort of about 70000 people aged 18–85 years, smoking was linked to worse verbal learning and memory in women than in men^22^. In another cohort of non-demented older adults aged ≥65 years, smoking was found to be associated with accelerated cognitive decline^23^. However, not all relevant studies point in the same direction of the relationship. In a cross-sectional baseline analysis of 16892 participants from the China Health and Retirement Longitudinal Study, researchers found that the number of pack-years was independently and positively associated with orientation, attention, and overall cognitive functioning in middle-aged and older Chinese adults^9^. However, in that study, researchers failed to adjust for important confounders such as body mass index. In another clinical trial, researchers found a U-shape relationship between smoking and cognition; while light nicotine use was associated with improved cognition, heavy smoking on a chronic and possibly acute basis impaired cognitive functioning^24^. Thus, the relationship between tobacco and cognitive functioning is complex.

The possible mechanisms that account for the association between tobacco exposure and worse cognitive functioning are indeed complex. Oxidative stress and inflammation are the primary pathophysiological mechanism explaining the long-term negative effects of tobacco exposure on cognitive function^25^. The uptake of the main harmful substances in cigarette smoke, such as particulate matter^26^, heavy metal ions^27^, reactive aldehydes^28^, and volatile organic compounds^29^, causes neuroinflammation and cerebral oxidative stress by activating microglia, and damages important pathways in the brain. In particular, the brain is specifically vulnerable to oxidative stress because of the large amount of oxidizable polyunsaturated fatty acids in membrane phospholipids as well as the high metabolic demand for oxygen^30-32^. The anterior frontal, medial and lateral temporal lobes and hippocampus are highly susceptible to cell damage mediated by oxidative stress^33^. In addition, current evidence has also shown that tobacco exposure may alter brain structure and neuronal function^25^. *In vitro* and animal studies have demonstrated that tobacco exposure leads to core amyloid synthesis and tau pathologies in brains, which are presumed to cause neurodegeneration, cell death, and cognitive decline^34,35^.

### Strengths and limitations

To our knowledge, this is the first study that examined the relationship between tobacco-specific nitrosamines and cognitive functioning in humans. A nationally representative sample of older adults was used as the study population. Thus, this study contributes to the literature and adds strong evidence to the negative cognitive effect of tobacco in humans. Moreover, since tobacco-specific NNAL captures both active and passive exposure to tobacco, it is more comprehensive than using self-report pack-years to assess individuals’ exposure to tobacco. Furthermore, tobacco-specific NNAL has a longer half-life than cotinine, the most common biomarker of cigarette exposure, and thus can better reflect cumulative tobacco exposure over time. Last, to lessen the possibility of confounding, a wide range of sociodemographic, lifestyle, mental health, and physical health covariates were adjusted for.

The limitation of this study is mainly the cross-sectional design which prevents us from assessing the temporal relationship between tobacco-specific nitrosamines and cognitive functioning. Moreover, although tobacco-specific NNAL has a relatively long half-life compared with cotinine, it still only measures a person’s recent exposure to tobacco and does not reflect his/her long-term exposure to tobacco. In addition, the excluded people due to missing data (n=112) and the included participants (n=1673) had ethnic, mental health, and lifestyle differences; therefore, selection bias may exist. Last, we may not have assessed all cognitive domains with only three cognitive tests. Future research should include longitudinal studies to examine the temporal relationship between tobacco-specific NNAL and the full cognitive domains in older adults, especially those from non-western countries.

## CONCLUSIONS

We found an independent and negative relationship between urinary tobacco-specific NNAL, a biomarker of tobacco exposure, and processing speed, sustained attention, and working memory in older adults. Given the negative effects of tobacco on cognitive functioning as well as multiple systems and organs in the human body^36^, clinicians and health educators should encourage older adults to avoid or reduce active and passive tobacco exposure. Clinicians should incorporate tobacco exposure into routine clinical assessments for older adults.

## Data Availability

The data that support the findings of this study are available on the NHANES website and can be accessed at https://wwwn.cdc.gov/nchs/nhanes/Default.aspx
