# Supplementary material for: Urinary tobacco-specific nitrosamine 4-(methylnitrosamino)-1-(3-pyridyl)-1-butanol (NNAL) and cognitive functioning in older adults: The National Health and Nutrition Examination Survey 2013–2014
Source: Tob Induc Dis. 2023 May 25;21:68. doi: 10.18332/tid/162368 (PMC10210584; doi:10.18332/tid/162368)
Supplement: Supplementary file 1 [file TID-21-68-s1.pdf]

Appendix. The characteristics of included and excluded participants due to missing data

Variables	Excluded (n=112)	Included (n=1673)	P Value
Age, years	72.6(7.1)	69.8(6.8)	**<0.001**
Sex, n (%)			0.159
Male	153(53.1%)	802(47.9%)	
Female	135(46.9)	871(52.1%)	
Race/ethnicity, n (%)			**0.026**
Mexican Americans	22(7.6%)	198(11.8%)	
Other Hispanics	47(16.3%)	145(8.7%)	
Non-Hispanic Whites	101(35.1%)	808(48.3%)	
Non-Hispanic Blacks	68(23.6%)	346(20.7%)	
Other	50(17.4)	176(10.5%)	
Education, n (%)			0.721
Below high school	100(34.8%)	441(26.3%)	
High school graduate	68(23.6%)	399(23.8%)	
Some college or above	119(41.3%)	831(49.7%)	
Body mass index , n (%)			0.587
<18.5 kg/m2	8(2.8%)	29(1.7%)	
18.5-24.9 kg/m2	71(24.7%)	415(24.8%)	
25.0-29.9 kg/m2	110(38.2%)	600(35.9%)	
≥30 kg/m2	93(32.3%)	604(36.1%)	
Depressive symptoms	6.2(6.4)	3.8(4.9)	**<0.001**
Physical activity, hours/week	0.7(3.3)	2.3(7.0)	**0.013**
Total cholesterol, mg/dL	174.1(42.0)	188.6(41.9)	**0.001**
Systolic blood pressure, mmHg	119.5(21.8)	116.5(16.2)	0.184
CERAD W-L immediate recall	15.9(6.1)	19.4(4.9)	**<0.001**
CERAD W-L delayed recall	5.1(2.6)	6.1(2.4)	**<0.001**
Animal Fluency Test	14.0(5.4)	16.6(5.5)	**<0.001**
Digit Symbol Substitution Test	36.8(15.5)	46.4(17.2)	**<0.001**

© 2023 Ge S. et al.
